# Effect of Phthalocyanines Substitution Pattern on Their Loading into Bacterial Cellulose Nanocrystals

**DOI:** 10.3390/molecules31081232

**Published:** 2026-04-08

**Authors:** Zeynel Şahin

**Affiliations:** Department of Metallurgical and Materials Engineering, Faculty of Technology, Marmara University, 34854 Istanbul, Türkiye; zeynel.sahin@marmara.edu.tr

**Keywords:** phthalocyanine, bacterial cellulose, nanomaterial, photosensitizer, singlet oxygen

## Abstract

Photodynamic therapy (PDT) has already gained immense attention in the anti-tumor field due to its low toxicity and non-invasiveness compared to traditional treatment methods. Therefore, the development of efficient photosensitizers is crucial for the advancement of photodynamic therapy. Although phthalocyanines (Pcs) have attracted huge attention as efficient photosensitizers, their clinical applications are hindered by poor solubility and a tendency to aggregate. Herein, two different Pcs that have different polarities were loaded into bacterial cellulose nanoparticles via non-covalent interactions. The aggregation behaviors and singlet oxygen production efficiencies were studied, as well as the influence of the Pc polarity on loading ratios. It was observed that octa-propylsulfonyl phthalocyanine ZnPc(SO_2_Pr)_8_, which has a more polar structure, loaded more on bacterial cellulose nanocrystal. Also, singlet oxygen generation efficiency of ZnPc(SO_2_Pr)_8_ was harmoniously increased with the loading ratio. The result indicated that both of the phthalocyanine/bacterial cellulose nanocrystal (Pc/BCNs) systems produced singlet oxygen and could be used as potential photosensitizers in PDT, especially ZnPc(SO_2_Pr)_8,_ due to the high loading ratio.

## 1. Introduction

Cancer is an increasing global health issue and the second-most common cause of death in the world, making it one of the most widespread civilization health issues [1,2]. Current cancer treatment methods, such as chemotherapy, radiotherapy, and surgical resection, are not perfect and face restrictions, including non-selectivity, resistance, severe side effects and high recurrence rates [3,4]. As an alternative to these therapeutic methods, photodynamic therapy (PDT) has gained significant attention as a promising cancer treatment. PDT is considered a non-invasive cancer treatment method that is based on the interactions of three basic components: photosensitizer (PS), molecular oxygen and light of a specific wavelength [5].

PSs usually display an intense absorption at a wavelength that depends on the chemical structure. Ideally, the same wavelength is used to excite the PS, which gets to its singlet excited state, then to the triplet state via intersystem crossing. The PS then gets back to its fundamental state either with non-radiative de-excitation, or by transferring energy to nearby molecules of oxygen, thereby converting it into singlet oxygen, which is responsible for cancer cell death via oxidative damages inducing apoptosis or necrosis. In addition to this so-called Type II mechanism, other reactive oxygen species (ROS) can be formed via an electron transfer (Type I mechanism). Using wavelengths of the phototherapeutic window to prevent damage to endogenous chromophores and ensure deeper penetration of the light into tissues is desired to avoid side effects and maximize the efficiency of the treatment.

In recent years, numerous PSs have been used by researchers and widely examined to explore their potential for PDT [6]. While porphyrin-based PSs are the most common PSs with current approval and ongoing clinical tests [7] (aminolevulinic acid/ALA enters this category as the in situ generated PS is actually PpIX), phthalocyanines are extremely promising in PDT because of their strong absorption in the phototherapeutic window and high singlet oxygen production efficiency for inactivation of cancer cells [8,9]. Phthalocyanines are synthetic macrocyclic compounds that resemble naturally occurring porphyrins and exhibit intense absorption at about 700 nm. Their optical properties can be tuned by changing the metal in the center or substituents on the macrocycle, making them highly attractive for PDT applications. Depending on the metal or semi-metal complexing the phthalocyanine macrocycle, axial substituents can also be introduced, increasing the structural versatility of phthalocyanine-based PSs and further modulating their properties while lowering their aggregation [10]. However, despite these superiorities, phthalocyanines have some restrictions when used in clinical applications, such as poor water solubility, aggregation, low tumor specificity, or undesired toxicity to healthy tissues [11,12]. To solve this problem and obtain the desired high photodynamic efficiency PS, encapsulating phthalocyanine in nanoparticles seems to be a promising approach [13]. Nanotechnology provides many possibilities, such as high loading capacity, extended circulation time, enhanced delivery, targeting capabilities and reduces toxicity to healthy tissues [14]. Nanoparticles are versatile materials that have unique properties like their size, surface characteristics, and ability to be functionalized with targeting compounds, making them ideal candidates for PDT [15,16,17].

Cellulose is one of the most abundant and renewable natural polymers, which is found in plant cell walls and also produced by algae, fungi, marine tunicates, and bacteria. Cellulose is a carbohydrate polymer that consists of long chains β-(1-4) linked D-glucose units [18,19]. Cellulose and its derivatives are used in many areas, such as the textile industry and the paper industry, as well as in cancer therapy, antibacterial applications, and bacterial imaging [20,21]. Bacterial cellulose (BC) is an excellent biopolymer produced by microorganisms and numerous bacteria such as *Agrobacterium*, *Rhizobium*, *Sarcina*, and *Gluconacetobacter*. Even if BC is similar to plant cellulose in terms of molecules, BC is gaining considerable attention due to its unique mechanical stability, high crystallinity, high purity (free of lignin, hemicellulose and pectin), large surface area, excellent permeability, high water-holding capacity, biocompatible and biodegradable [22,23]. Therefore, it has been considered as a basic source for the production of cellulose nanocrystals, and synthesized by acid hydrolysis, enzyme and ionic liquid. Among these methods, bacterial cellulose nanocrystal (BCNs) is obtained using inorganic acids, such as hydrochloric acid (HCl), sulfuric acid (H_2_SO_4_) and a mixture of HCl and H_2_SO_4_ [24]. In particular, BCNs prepared by H_2_SO_4_ endow with anionic sulfate half-ester groups and this gives rise to highly negative charge on their surface [25]. Due to the negative-charges of BCNs, they can prevent their aggregation and obtain a well-dispersed nanocrystal suspension. BCNs is considered to be amphiphilic [26] and organic and inorganic compounds incorporated in their structure [27,28].

In this study, two different types of zinc phthalocyanines substituted with propylsulfanyl and propylsulfonyl groups were loaded into bacterial cellulose nanocrystals with different rates of non-covalent interactions. Molecular structure, the morphology and size, loading amount, aggregation behavior in solutions and singlet oxygen generation efficiency of the Pc/BCNs were studied using spectroscopic methods.

## 2. Results and Discussion

### 2.1. Synthesis of Phthalocyanines

**Figure 1:**
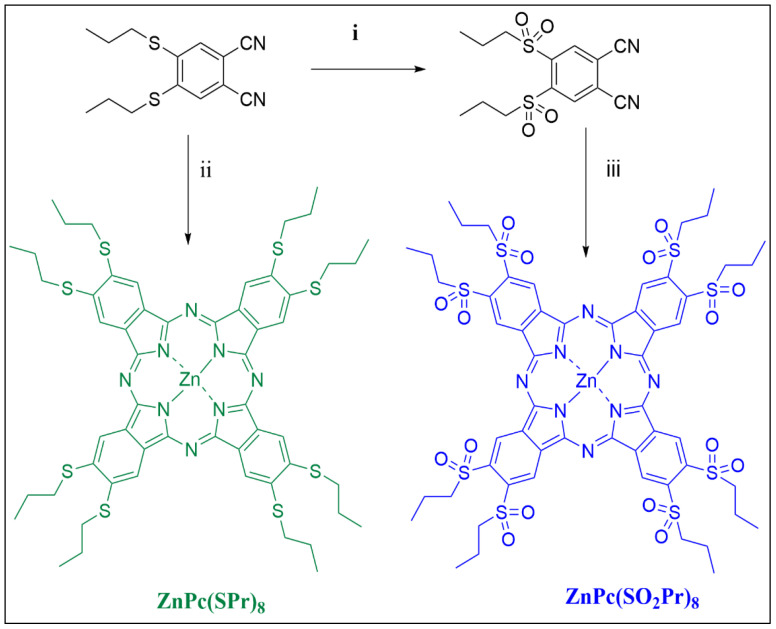
Synthesis phthalocyanines ZnPc(SPr)_8_ and ZnPc(SO_2_Pr)_8_: (i) Acetic acid, H_2_O_2_. (ii) n-Pentanol, DBU, Zn(OAc)_2_, 18 h reflux. (iii) o-Dichlorobenzene, DMF, Zn(OAc)_2_, 18 h reflux.

The synthesis of target phthalocyanines was depicted in Figure 1. The details for preparing the 4,5-bis(propylsulfanyl)phthalonitrile, 4,5-bis(propylsulfonyl)phthalonitrile, and ZnPc(SO_2_Pr)_8_ were given in the literature [29,30]. The ZnPc(SPr)_8_ phthalocyanine was obtained by cyclotetramerization of 4,5-bis(propylsulfanyl)phthalonitrile in the presence of Zn(OAc)_2_ and DBU in pentanol under N_2_ atmosphere. Structure of the ZnPc(SPr)_8_ was confirmed by FT-IR, ^1^H-NMR, MALDI and UV-vis spectroscopic techniques. The IR spectrum of the ZnPc(SPr)_8_ was easily confirmed by the structure that specific -C≡N stretching (~2200 cm^−1^) of the phthalonitriles, which disappeared after the conversion into ZnPc(SPr)_8_ compound (Figure S1) [31]. In the ^1^H-NMR spectra of ZnPc(SPr)_8_, aromatic and aliphatic protons are compatible with the structure as expected. In the mass spectra, the molecular ion peak of the ZnPc(SPr)_8_ was shown at 1168.223 as expected. The UV-vis spectroscopy is the most used method for the characterization of the phthalocyanines. Phthalocyanines have similar absorption spectra in the UV-vis regions. The UV region exhibits at 300–350 nm (B band) and the other in the visible region at 600–700 nm (Q-band) [32,33]. The UV-vis spectra of the ZnPc(SPr)_8_ confirmed the structure that exhibited specific Q and B bands at 693 and 365 nm.

### 2.2. Synthesis of Bacterial Cellulose (BC) and Bacterial Cellulose Nanocrystal (BCNs)

*Gluconacetobacter xylinus* was used to produce BC pellicles. The BC was produced using the Hestrin and Schramm (HS) culture medium prepared in distilled water that consists of 2.0 wt% D-glucose, 0.5 wt% peptone, 0.5 wt% yeast extract, 0.27 wt% disodium hydrogen phosphate, and 0.115 wt% citric acid. The pH value of the medium was adjusted to 5.0–6.0 with acetic acid [34,35], followed by 80 °C sterilization for 20 min and allowed to cool to room temperature. BC pellicles production was performed in static conditions at ~25 °C for 7 days. At the end of the production process, the BC pellicles were purified by 0.1 M NaOH for 24 h, removing cell culture and other impurities. The BC pellicles were washed several times with deionized water for the neutralized and sterilized using an autoclave at 120 °C for 20 min. The purified BC membranes were cut into small pieces and homogenized for 15 min using a household homogenizer to obtain a homogeneous bacterial cellulose suspension. The BC solution was centrifuged at 6000 rpm for 10 min and then dried in oven at 40 °C for one night for further use.

BCNs were synthesized by sulfuric acid hydrolysis of the dried BC similar to the given literature, with some modifications [36]. In brief, 0.3 g oven-dried BC was mixed with 20 mL 60% (*w*/*w*) H_2_SO_4_ at 45 °C for 3 h under continuous magnetic stirring (400 rpm). To stop the hydrolysis reaction, the mixture was diluted five-fold with ultrapure water. Afterwards, the suspension was centrifuged three times at 6000 rpm for 10 min to separate the crystal and remove excess acid. The precipitate was dialyzed against deionized water for 4 days using a molecular weight cutoff 3500 dialyzing membrane to remove residual H_2_SO_4_ and other low molecular weight impurities. The dialysis solution was centrifuged and obtained 1.4% (*w*/*w*) BCNs aqueous solution. A particular amount of the BCNs solutions was dried to calculate the BCNs amount in the solution, and the rest of the BCNs aqueous solution was stored at 4 °C. Based on the dried BCNs, the yield was 70%.

### 2.3. Preparation of Phthalocyanine-Loaded BCNs Solutions

**Figure 2:**
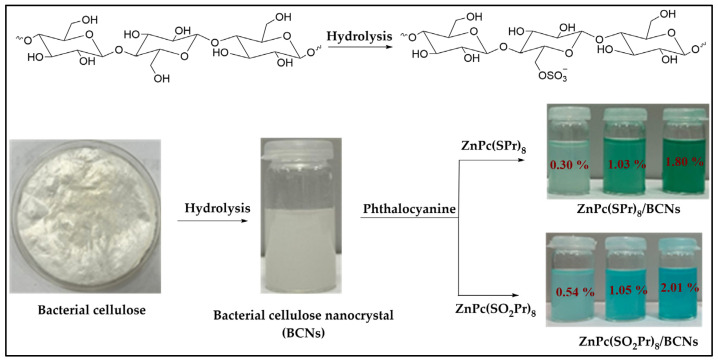
Pictures of the preparation of the phthalocyanines-BCNs.

Three different solutions of both ZnPc(SPr)_8_/BCNs/BCNs and ZnPc(SO_2_Pr)_8_/BCNs were prepared in DMF by adding different amounts of phthalocyanine to BCNs suspension and route as illustrated in Figure 2. Firstly, phthalocyanine stock solutions were prepared by dissolving 0.0043 g of ZnPc(SPr)_8_ and 0.0051 g of ZnPc(SO_2_Pr)_8_ of powder in 10 mL DMF that concentration of 3.67 × 10^−4^ and 3.58 × 10^−4^, respectively. To prepare the phthalocyanines/BCNs (Pcs/BCNs) solutions, a particular amount of BCNs (9.4 mg for ZnPc(SPr)_8_ and 11.2 mg for ZnPc(SO_2_Pr)_8_) was dispersed in 2 mL of water, and then the phthalocyanine solution was added to in BCNs suspension. Using the phthalocyanine stock solutions, three different Pcs/BCNs solutions were prepared for each sample.

Briefly, for the preparation of ZnPc(SPr)_8_/BCNs stock solutions, 131 μL (56.3 μg), 392 μL (168.9 μg), and 654 μL (281.5 μg) solutions of the ZnPc(SPr)_8_ in DMF were added to BCNs suspensions under magnetic stirring (500 rpm) and stirred for another 4 h. The ZnPc(SPr)_8_/BCNs particles were obtained by centrifugation at 6000 rpm for 20 min at room temperature, washed three times with DMF to remove free (non-loaded) ZnPc(SPr)_8_. Then, the total volume of the ZnPc(SPr)_8_/BCNs stock solutions was adjusted to 8 mL by adding DMF and stored in a refrigerator at 4 °C.

The other three stock solutions of the ZnPc(SO_2_Pr)_8_/BCNs were prepared using the same method, by adding 134 μL, 402 μL, and 699 μL solution of the ZnPc(SO_2_Pr)_8_ to BCNs suspension, respectively.

#### Phthalocyanine Loading Ratio

The Pc content in the BCNs was calculated as the difference between the initial phthalocyanine amount added in the BCNs and the non-loaded phthalocyanine in the washing DMF solution. The concentration of the non-loaded phthalocyanines was determined using the calibration curve of the phthalocyanine. The calibration curve was obtained by measuring the absorption spectra in the range of 2–10 μM concentration of the phthalocyanines in DMF, and UV-vis spectra are given in Figures S2 and S3. The loading rate of the Pc was calculated by following Equation (1);

(1) $$Loading\;ratio\left(\%\right)=\frac{W_{i}-W_{r}}{W_{BCNs}}\times 100$$

where W_i_ represents the initial weight of the Pc added in the BCNs suspension, W_r_ represents the residual weight of Pc in washing DMF solution, and W_BCNs_ represents the weight of the BCNs in suspension [37].

The calculated Pc loading ratios (wt%) for ZnPc(SPr)_8_/BCN are 0.30, 1.03, and 1.80, and for ZnPc(SO_2_Pr)_8_/BCNs are 0.54, 1.05, and 2.01, respectively. The suspension concentration (mg/mL) of the Pc/BCNs sample was calculated by following Equation (2);

(2) $$Suspen.concentration\;(\frac{mg}{mL})=\frac{W_{P_{c}}+{\;W}_{BCNs}}{V_{T}}$$

where W_Pc_ represents the weight of the Pc loaded in the BCNs, W_BCNs_ represents the weight of the BCNs in suspension, and V_T_ represents the total volume of suspension.

The suspension concentration for ZnPc(SPr)_8_/BCN are 1.18 mg/mL, 1.19 mg/mL, and 1.20 mg/mL, and for ZnPc(SO_2_Pr)_8_/BCNs are 1.41 mg/mL, 1.42 mg/mL, and 1.43 mg/mL, respectively. The results indicated that the loading rates for both Pcs increased in accordance with the amount of phthalocyanine added. However, when the Pcs were compared with each other, the more polar ZnPc(SO_2_Pr)_8_ had higher loading rates in each of the three samples. The Pc/BCNs samples were abbreviated as ZnPc(SPr)_8_/BCN-1, ZnPc(SPr)_8_/BCN-2, ZnPc(SPr)_8_/BCN-3 and ZnPc(SO_2_Pr)_8_/BCN-1, ZnPc(SO_2_Pr)_8_/BCN-2, ZnPc(SO_2_Pr)_8_/BCN-3 according to Pc loading ratios (wt%).

### 2.4. FT-IR Analysis of BC, BCNs and Pc/BCNs Sample

**Figure 3:**
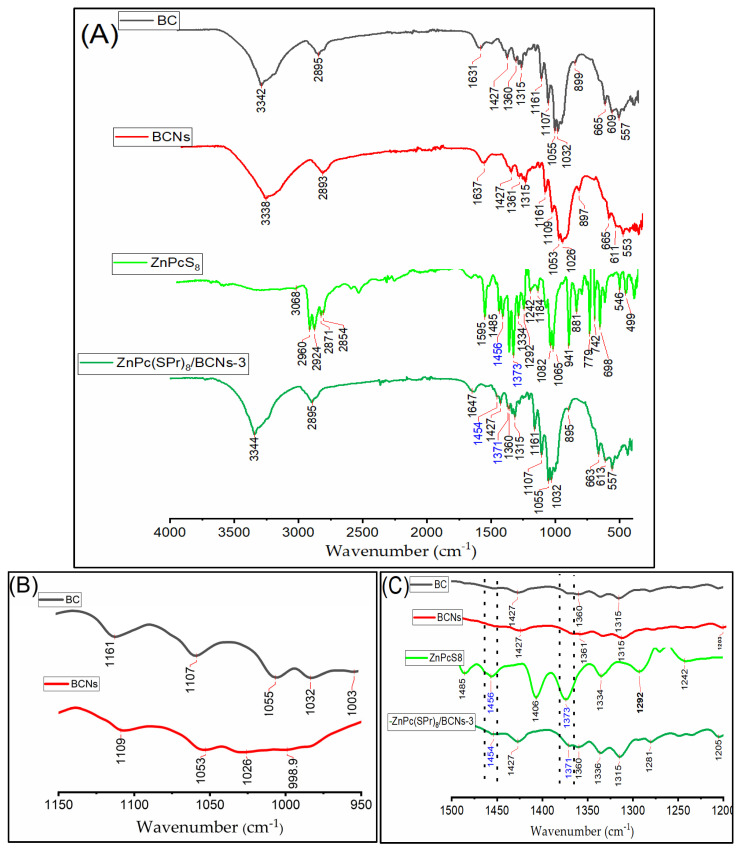
(**A**) FT-IR spectrum of BC, BCNS, ZnPc(SPr)8 and ZnPc(SPr)8/BCNs-3. (**B**) Enlarged FT-IR spectra of BC and BCNs. (**C**) Enlarged FT-IR of BC, BCNS, ZnPc(SPr)8 and ZnPc(SPr)8/BCNs-3.

The FT-IR spectra of BC, BCNs, ZnPc(SPr)_8_ and ZnPc(SPr)_8_/BCN-3 are presented in Figure 3. The FT-IR spectra of the BC and BCNs showed similar vibration bands, with the strong and broad absorption band at 3000–3500 cm^−1^ attributed to O-H stretching vibrations [38]. In these regions, the BCNs spectrum is broader than the BC spectrum. The O-H band of the ZnPc(SPr)_8_/BCN-3 is narrowest than BC and BNCs, and its most distinctive features. These difference in the O-H band indicate that the hydroxyl group ratio on the samples surface and the interaction of intermolecular and intramolecular are different between samples [39]. Further, the absorption bands about 2890 cm^−1^ were assigned to the C-H bonds stretching of -CH_2_ and -CH_3_ groups. The peaks around 1646–1637 cm^−1^ are attributed to O-H bending of adsorbed water. The peaks at around 1160 and 1054 cm^−1^ are assigned to -O-H stretching and -OH wagging in the C-O-C pyranose ring [40]. Also, the decrease in the intensity of bands at 1100–1000 cm^−1^ regions was confirmed in the BC dehydrations that hydroxyl groups substituted with sulfate groups (Figure 3B) [39].

Similar FT-IR spectra were observed in Pcs/BCNs samples, all the peaks in the BCNs spectrum were founded in Pcs/BCNs samples with a slightly different intensity and shifts. But the strong and wider bands of BCNs suppressed the Pcs peaks in all the Pcs/BCNs samples. For example, there is no difference between the BNCs and ZnPc(SPr)_8_/BCN-3 spectra, and they cannot be distinguished by the FT-IR spectrum. However, 1456 and 1373 cm^−1^ peaks in the ZnPc(SPr)_8_ spectrum are shown as weak peaks at 1454 and 1371 cm^−1^ in the ZnPc(SPr)_8_/BCN-3 sample, indicating that the ZnPc(SPr)_8_ molecule was incorporated into the BCNs (Figure 3C).

The FT-IR spectra of ZnPc(SPr)_8_/BCN-(1 and 2), ZnPc(SO_2_Pr)_8_, ZnPc(SO_2_Pr)_8_/BCNs-(1 and 2), superimposed spectra of the BC, BCNs, ZnPc(SO_2_Pr)_8_ and (ZnPc(SO_2_Pr)_8_/BCNs-3 were represented Supplementary Information Figures S4–S9.

### 2.5. Scanning Electron Microscopy

**Figure 4:**
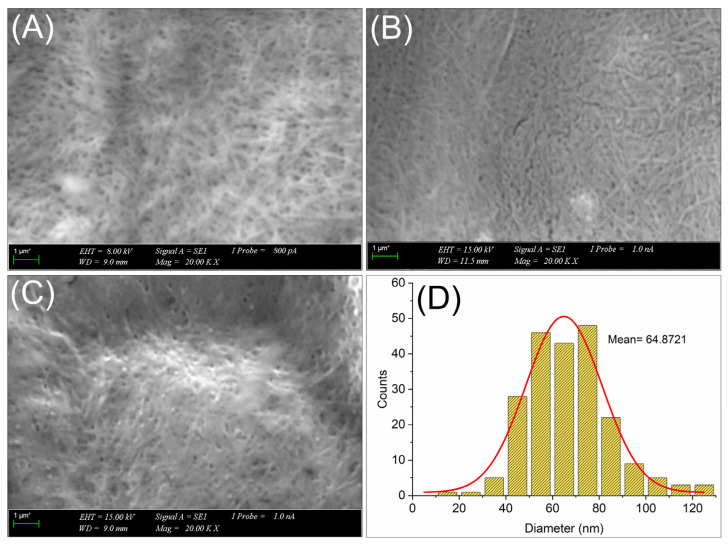
SEM images of BCNs (**A**), ZnPc(SPr)_8_/BCNs-3 (**B**), ZnPc(SO_2_Pr)_8_/BCNs-3 (**C**) and diameter distribution curve of BCNs (**D**).

BC has a distinct ribbon-like 3D network structure with a diameter of around 100 nm and a length of around 100 μm. Depending on the carbon source, isolation method and hydrolysis conditions (acid concentration, reaction temperature, reaction time), the dimensions of the prepared BCNs are about 10–50 nm in diameter and 100–1500 nm in length [22,41]. The SEM images of BCNs, ZnPc(SPr)_8_/BCNs-3, ZnPc(SO_2_Pr)_8_/BCNs-3 and the diameter distribution curve of BCNs are shown in Figure 4. The acid hydrolysis BCNs exhibited a typical rod-like structure or interconnected microfibrils, as described by previous studies [36]. After loading Pcs molecules on the BCNs, the morphology became more compact, decreased the porous networks, and appeared to have some localized cluster regions as a result of the tight packing of fibril networks. This can be explained by the intermolecular interactions of Pcs molecules loaded onto the BCNs. These intermolecular interactions caused agglomeration of the fibrils, and samples exhibited higher fiber dimensions. The (ZnPc(SO_2_Pr)_8_/BCNs-3 more polar than ZnPc(SPr)_8_/BCNs-3), a more compact and less porous structure, was observed on the surface (Figure 4C). The diameter distribution analysis of the BCNs revealed a population with an average diameter of 64.8 nm (Figure 4D), which is consistent with the previous studies [42,43]. Because of the highly entangled and aggregated structure of the fibrils, fiber length of the BCNs could not be measured.

### 2.6. DLS and Zeta Potential

Dynamic light scattering (DLS) is an important technique in determining the hydrodynamic size distribution of non-spherical BCN nanoparticles. The hydrodynamic size distribution of acid-hydrolyzed BCNs was investigated in previous studies, which exhibited polydisperse size distributions from 22 to 5600 nm. Depending on the reaction parameters such as time, temperature and acid concentration (acid or mixture of acids), two or three peaks were observed on the DLS spectra with different size distributions. Additionally, the zeta potential values of BCNs were reported range from −11 to −53 mV [44].

**Figure 5:**
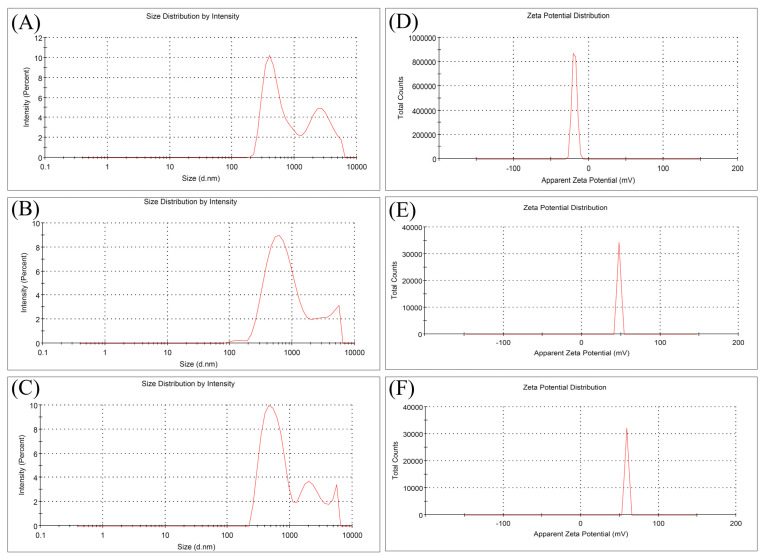
Particle size analysis and zeta potential of BCNs (**A**–**D**), ZnPc(SPr)_8_/BCNs-3 (**B**–**E**) and ZnPc(SO_2_Pr)_8_/BCNs-3 (**C**–**F**), respectively.

In this study, two and three different peaks were observed in the hydrodynamic size distribution curves of BCNs and Pc/BCNs (Figure 5A–C). As shown in Figure 5A, BCNs have two distinct particle size distributions with an average hydrodynamic diameter of 668.4 nm and a polydispersity index (PDI) of 0.417. The primary peak is around 300 nm and the secondary peak is around 1500 nm, indicating clustering and a heterogeneous system. Fallowing the loading of Pc molecules on BCNs, slightly changes in size distributions were observed. The ZnPc(SO_2_Pr)_8_/BCNs-3 sample (Figure 5C) has a relatively narrow size distribution compared to ZnPc(SPr)_8_/BCNs-3 (Figure 5B), their average hydrodynamic diameters are 749.6 nm (PDI = 0.494) and 700.8 nm (PDI = 0.416), respectively.

Zeta potential measurements were conducted to evaluate the surface charge and colloidal stability of BCNs and Pc/BCNs samples. BCNs exhibited a zeta potential of −16.6 mV (Figure 5D), indicating poor colloidal stability. In contrast, ZnPc(SPr)_8_/BCNs-3 and ZnPc(SO_2_Pr)_8_/BCNs-3 showed significantly higher zeta potential values of +47.5 mV (Figure 5E) and 59.1 mV (Figure 5F), respectively. The Pcs molecules used in this study are inherently non-cationic, but their zeta potentials turned into a net positive value after being absorbed onto the surface of BCNs. This change can be explained by the tight binding of Pc molecules to BCNs and the formation of layers that cause a positive zeta potential. These values indicate that Pc molecules were successfully loaded on BCNs. The higher zeta potential of ZnPc(SO_2_Pr)_8_/BCNs-3 suggests stronger electrostatic repulsion and improved colloidal stability. From the above results, the ZnPc(SO_2_Pr)_8_/BCNs-3 sample possessed a small particle size and a more homogeneous particle size distribution.

### 2.7. UV-Vis Absorption and Fluorescence Spectra

**Figure 6:**
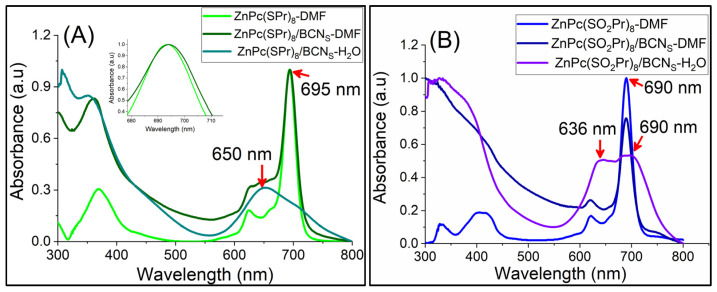
(**A**) Normalized UV-vis absorption spectra of ZnPc(SPr)_8_ in DMF, ZnPc(SPr)_8_/BCNs-3 in DMF, and water. (**B**) Normalized UV-vis absorption spectra of ZnPc(SO_2_Pr)_8_ in DMF and ZnPc(SO_2_Pr)_8_/BCNs-3 in DMF and water.

**Figure 7:**
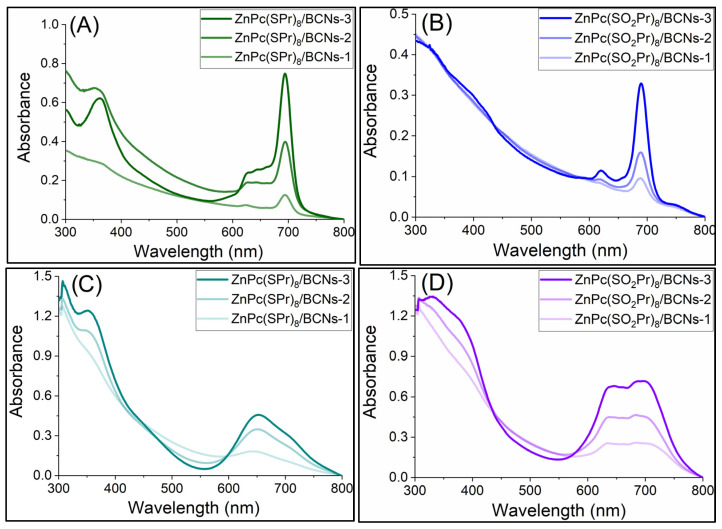
Absorption spectra of ZnPc(SPr)_8_/BCNs and of ZnPc(SO_2_Pr)_8_/BCNs in DMF (**A**,**B**) and in water (**C**,**D**), respectively.

The UV-vis absorption spectra of the Pcs in DMF, and of Pc/BCNs samples in DMF and in water are shown in Figure 6 and Figure 7. The absorption spectra of the Pc/BCNs samples were recorded in DMF and in water. All of the Pc/BCNs samples exhibited the same typical absorption spectra of the related monomeric Pc. There were no significant shifts in the Q-bands of the ZnPc(SPr)_8_/BCNs materials compared to ZnPc(SPr)_8_ alone in DMF (Figure 6A) [45]. Similarly, and as seen in Figure 6B, no shifts were observed in the Q-band of the ZnPc(SO_2_Pr)_8_/BCNs and of ZnPc(SO_2_Pr)_8_ in DMF. In water, the Q-bands of the Pc/BCNs samples are blue-shifted and broadened as compared to the absorption of the same samples in DMF, indicating that the Pcs are slightly aggregated with a H-type aggregation [46]. The Q-band maximum of the ZnPc(SPr)_8_/BCNs exhibits a 45 nm hypochromic shift in water compared to DMF (Figure 6A). For the ZnPc(SO_2_Pr)_8_/BCNs in water, the blue shift is only partial and a split Q-band is observed, with a shoulder at 636 nm but remaining absorption at 690 nm, indicating that the Pc is less aggregated onto BCNs when in water. As the Pc loading ratio increased in the BCNs, the intensity of Q-band also increased harmoniously for both Pc/BCNs samples (Figure 7). The linearity of the maximum absorption of each material in water (ZnPc(SPr)_8_/BCNs and ZnPc(SO_2_Pr)_8_/BCNs) was checked at different concentrations (Figure S10), showing that the Beer–Lambert law was followed.

**Figure 8:**
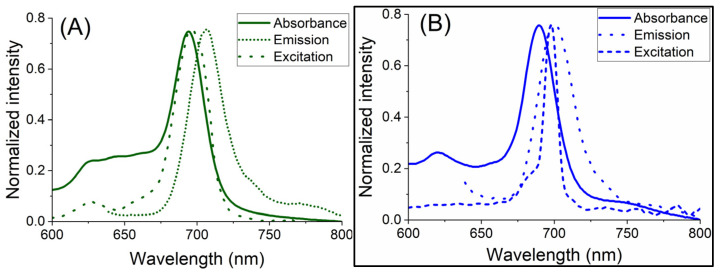
The UV-vis absorption, emission and excitation spectra of (**A**) ZnPc(SPr)_8_/BCNs-3 and (**B**) ZnPc(SO_2_Pr)_8_/BCNs-3 in DMF. Excitation wavelength: 625 nm for both samples.

The superimposed absorption, emission and excitation spectra of Pc/BCNs-3 samples are shown in Figure 8. The fluorescence excitation spectra of the samples are similar to absorption spectra and are a mirror image of the emission spectra. Slight differences between absorption and excitation spectra of ZnPc(SO_2_Pr)_8_/BCN-3 (Figure 8B) could be due to different equipment used [47,48]. In the absorption and emission spectra of ZnPc(SPr)_8_/BCNs-3 were observed at 695 nm and 706 nm, respectively, giving a Stokes shift of 11 nm. Similarly, the absorption and emission spectra of ZnPc(SO_2_Pr)_8_/BCNs-3 were observed at 689 nm and 699 nm, and a 10 nm Stokes shift was seen.

### 2.8. Singlet Oxygen Generation Assay

**Figure 9:**
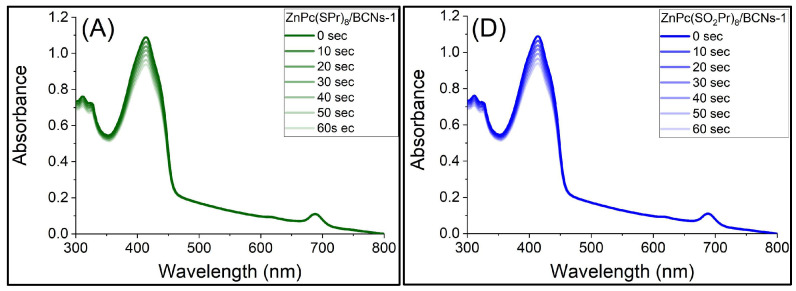

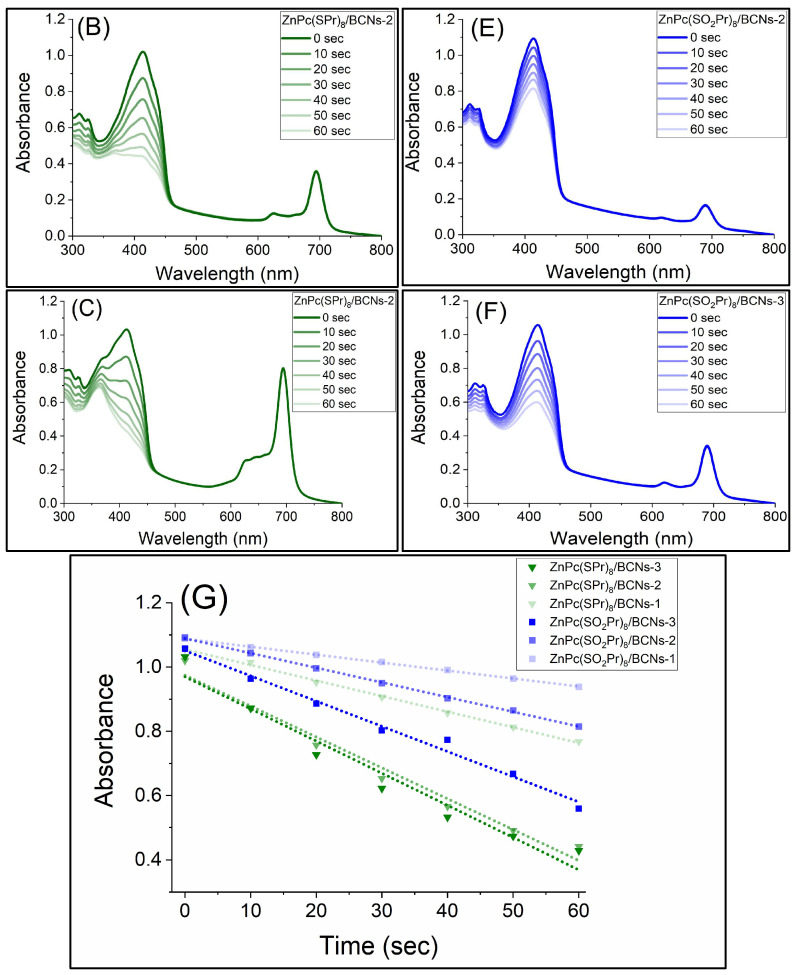
Absorption spectra of DPBF in DMF and after different irradiation durations (0–60 s), in the presence of ZnPc(SPr)_8_/BCNs-1 (**A**), ZnPc(SPr)_8_/BCNs-2 (**B**), ZnPc(SPr)_8_/BCNs-3 (**C**), ZnPc(SO_2_Pr)_8_/BCNs-1 (**D**), ZnPc(SO_2_Pr)_8_/BCNs-2 (**E**) and ZnPc(SO_2_Pr)_8_/BCNs-3 (**F**). Superimposed decay curves for relative absorption of DPBF at 413 nm with different irradiation durations (0–60 s) in the presence of the studied materials (**G**).

The efficient production of singlet oxygen (^1^O_2_) is an important prerequisite for photodynamic therapy. To evaluate the ^1^O_2_ generation efficacy of the Pc/BCNs samples, 1,3-diphenyl isobenzofuran (DPBF) was used as a singlet oxygen quencher. The reaction between DPBF and ^1^O_2_ is irreversible, and the generation of ^1^O_2_ by Pc/BCNs samples was monitored by the decrease in the maximum absorption peak of DPBF at 413 nm [49,50]. The typical absorption intensity change in DPBF after increasing irradiation durations (0–60 s) is shown in Figure 9A–F. When the Pc/BCNs sample was irradiated by light, the intensity of the absorption peak of DPBF continuously decreased as the irradiation time increased, indicating efficient ^1^O_2_ generation. After the 60 s exposure time, the absorbance value of the DPBF in the presence of ZnPc(SPr)_8_/BCNs-3 and ZnPc(SO_2_Pr)_8/_BCNs-3 decreased continually, decreasing 58% and 43% the initial value, respectively. The observation of the sharp decrease in DPBF absorption peaks revealed a remarkable photosensitizing capacity. To compare the singlet oxygen generation ability of each material, the superimposed decay curves for the relative absorption of DPBF at 413 nm with different irradiation durations have been plotted in Figure 9G. One can see the effect of the increasing loading of ZnPc(SO_2_Pr)_8_ in the BCNs. The singlet oxygen generation is more efficient with ZnPc(SPr)_8_ containing materials, and the experiments show that a maximum loading effect is reached as no significant difference is observed for ZnPc(SPr)_8_/BCNs-2 and ZnPc(SPr)_8_/BCNs-3.

## 3. Materials and Methods

### 3.1. Materials

All chemicals and reagents were obtained commercially and used without additional purification. Dichloromethane (DCM), 1-pentanol, 1,8-diazabicyclo [5.4.0]undec-7-ene (DBU), acetone, H_2_SO_4_, dimethylformamide (DMF), 1,3-diphenylisobenzofuran (DPBF), disodium hydrogen phosphate (M_w_ = 141.96 g/mol), glucose (M_w_ = 180.16 g/mol), citric acid and glacial acetic acid were purchased as reagent grade from Aldrich (Burlington, MA, USA) or Merck (Darmstadt, Germany). Column chromatography was performed on silica gel (230–400 mesh, 60 A).

Infrared spectra were recorded on an FT-IR-4700 spectrometer (JASCO Corporation, Hachioji, Japan) with an attenuated total reflection (ATR) accessory featuring a diamond/ZnSe plate. Samples were deposited on the ATR module and recorded in the range of 4000–650 cm^−1^. UV-visible absorption spectra were recorded with a Shimadzu UV-2600 spectrophotometer (Kyoto, Japan) using a 1 cm path length cuvette between 300 and 800 nm. Stock solutions of Pc and Pc/BCNs were prepared in spectrophotometric-grade DMF and ultrapure water.

Steady-state fluorescence excitation and emission spectra were recorded by using a Varian Cary Eclipse spectrofluorometer (New York, NY, USA) using a 1 cm path length cuvette at room temperature. Mass spectra were obtained on a MALDI (matrix-assisted laser desorption ionization) BRUKER Microflex LT (Bremen, Germany) using dithranol (DIT) as the matrix. Particle size of the nanocrystal was analyzed by SEM (ZEISS, Jena, Germany) and measured using ImageJ software (v1.47, NIH, Bethesda, MD, USA). 4,5-Bis(propylsulfanyl)phthalonitrile, 4,5-bis(propylsulfonyl)phthalonitrile, and 2,3,9,10,16,17,23,24-octakis(propylsulfonyl)phthalocyaninato zinc (ll) ZnPc(SO_2_Pr)_8_ were synthesized according to the given literature [29,30].

### 3.2. Synthesis of 2,3,9,10,16,17,23,24-Octakis(propylsulfanyl)phthalocyaninato Zinc (ll), ZnPc(SPr)_8_

The 4,5-bis(propylsulfanyl)phthalonitrile (2.5 g, 9.05 mmol), anhydrous Zn(OAc)_2_ (0.807 g, 4.39 mmol) and a catalytic amount of DBU were stirred overnight in refluxing pentanol (b.p. 138 °C) under argon atmosphere. The reaction mixture was allowed to cool to room temperature and poured onto an ethanol–water (1:1) mixture. The solid mixture was filtered, dissolved in DCM and dried on sodium sulfate. The filtrate was evaporated and the solid product was isolated by column chromatography on silica gel using a mixture of DCM/ethanol (100/1). The final product was washed 3 times with hot ethanol and dried under vacuum. Yield: 12% (22 mg). FT-IR (ν, cm^−1^): 3068, 2960, 2923, 2854, 1594, 1484, 1456,1373, 1334, 1242, 1184, 1082, 1064, 941, 779, 742, 698. 1H NMR (500 MHz, CDCl_3_) δ, ppm: 6.60 (8 H, aromatic), 2.54 (16 H, m, SCH_2_CH_2_CH_3_), 1.29 (16 H, m, SCH_2_CH_2_CH_3_), 0.88 (24 H, m, SCH_2_CH_2_CH_3_). MALDI- TOF MS (DIT): *m*/*z*: 1168.223; calculated for C_56_H_64_N_8_S_8_Zn, 1168.231.

### 3.3. Dynamic Light Scattering (DLS) and Zeta Potential (ZP)

DLS analysis of the average particle sizes and zeta potentials of BCNs and Pc/BCNs were measured using a Zetasizer Nano ZS (Malvern Instruments, Malvern, UK). For this purpose, BCNs ZnPc(SO2Pr)_8_/BCNs-3 and ZnPc(SPr)_8_/BCNs-3 samples were used for measurement without any dilutions. The measurement was performed three times for each sample.

### 3.4. Singlet Oxygen (^1^O_2_) Generation

To evaluate ^1^O_2_ generation ability of the **Pc**/BCNs samples, DPBF was used as a ^1^O_2_ trapping reagent in DMF solution. The solution of the Pc/BCNs suspension containing DPBF was prepared in the dark and irradiated in the Q-band region. In a typical experiment, the DPBF solution was mixed with 3 mL Pc/BCNs suspension in a quartz cuvette. A 300 W-quartz lamp (Willoughby, OH, USA) was used as a light source, a water filter and a 600 nm glass cutoff filter were used to filter off ultraviolet and infrared radiations. A 700 nm filter, allowing us to pass the appropriate wavelength light for the Q-band region was placed before the Pc/BCNs sample.

## 4. Conclusions

In summary, a series of ZnPc(SPr)_8_/BCNs and ZnPc(SO_2_)_8_/BCNs photosensitizers were successfully prepared by loading the octapropylsulfanyl-substituted ZnPc(SPr)_8_ and octapropylsulfonyl-substituted ZnPc(SO_2_Pr)_8_ into bacterial cellulose nanocrystals. After phthalocyanines were loaded inside the bacterial cellulose nanoparticle, the percent of loading ratio, aggregation behavior, and ability of singlet oxygen generation were investigated. Equal amounts (Pc/BCN ratio, wt%) of ZnPc(SPr)_8_ and ZnPc(SO_2_Pr)_8_ were added to BCNs suspensions, to study the effect of the loading ratio, allowing us to show that it was higher for ZnPc(SO_2_Pr)_8_ compared to ZnPc(SPr)_8_ in all samples. It can be deduced that ZnPc(SO_2_Pr)_8_, which has similar polar groups to BCNs in its structure, interacts strongly with BCNs. Generation of singlet oxygen was confirmed in all Pc/BCN samples, but the ZnPc(SO_2_Pr)_8_/BCNs-(1-3) samples produced singlet oxygen consistent with increasing loading rates, while ZnPc(SPr)_8_-based materials reached a maximum effect of the loading and were overall more efficient to generate singlet oxygen. Overall results show that phthalocyanine-bacterial cellulose materials can be used as a singlet oxygen source for photodynamic therapy. These materials are being tested in ongoing biological experiments to assess their efficiency in antimicrobial applications.

## Data Availability

Data are contained within the article and Supplementary Materials.

## Supplementary Materials

The following supporting information can be downloaded at: https://www.mdpi.com/article/10.3390/molecules31081232/s1, Figure S1. FT-IR spectrum of ZnPc(SPr)_8_. Figure S2. UV-vis spectrum of ZnPc(SPr)_8_ in DMF. Figure S3. UV-vis spectrum of ZnPc(SO_2_Pr)_8_ in DMF. Figure S4. FT-IR spectrum of ZnPc(SPr)_8_/BCNs-1. Figure S5. FT-IR spectrum of ZnPc(SPr)_8_/BCNs-2. Figure S6. FT-IR spectrum of the ZnPc(SO_2_Pr)_8_. Figure S7. FT-IR spectrum of ZnPc(SO_2_Pr)_8_/BCNs-1. Figure S8. FT-IR spectrum of ZnPc(SO_2_Pr)_8_/BCNs-2. Figure S9. Superimposed FT-IR spectra of BC, BCNs, ZnPc(SO_2_Pr)_8_ and ZnPc(SO_2_Pr)_8_/BCNs-3. Figure S10. UV-vis spectra of the Pc/BCNs samples at different concentration (mg/mL) in water.
