# Supplementary material for: Effect of Phthalocyanines Substitution Pattern on Their Loading into Bacterial Cellulose Nanocrystals
Source: Molecules. 2026 Apr 8;31(8):1232. doi: 10.3390/molecules31081232 (PMC13118783; doi:10.3390/molecules31081232)
Supplement: Supplementary file 1 [file molecules-31-01232-s001.zip › molecules-4166801-supplementary.pdf]

SUPPORTING INFORMATION for

Effect of Phthalocyanines Substitution Pattern on Their Loading into Bacterial Cellulose Nanocrystals

Zeynel Şahin

Department of Metallurgical and Materials Engineering, Faculty of Technology, Marmara University, 34854 Istanbul, Türkiye

Content

**Figure S1**. FT-IR spectrum of **ZnPc(SPr)$_8$**	2
**Figure S2**. UV-vis spectrum of **ZnPc(SPr)$_8$** in DMF	2
**Figure S3**. UV-vis spectrum of **ZnPc(SO$_2$Pr)$_8$** in DMF	3
**Figure S4.** FT-IR spectrum of **ZnPc(SPr)$_8$/BCNs-1**	3
**Figure S5.** FT-IR spectrum of **ZnPc(SPr)$_8$/BCNs-2**	4
**Figure S6.** FT-IR spectrum of the **ZnPc(SO$_2$Pr)$_8$**	4
**Figure S7.** FT-IR spectrum of **ZnPc(SO$_2$Pr)$_8$/BCNs-1**	5
**Figure S8.** FT-IR spectrum of **ZnPc(SO$_2$Pr)$_8$/BCNs-2**.	5
**Figure S9.** Superimposed FT-IR spectra of **BC**, **BCNs, ZnPc(SO$_2$Pr)$_8$** and **ZnPc(SO$_2$Pr)$_8$/BCNs-3**.	6
**Figure S10.** UV-vis spectra of the **Pc/BCNs** samples at different concentration (mg/mL) in water.	7

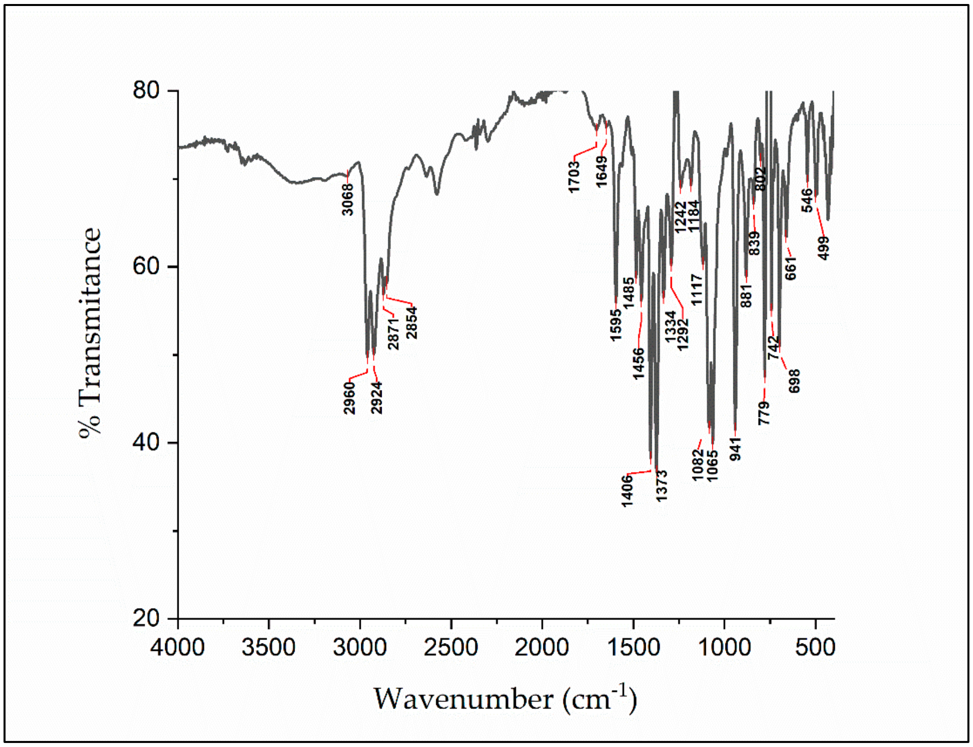

Figure S1. FT-IR spectrum of **ZnPc(SPr)₈**

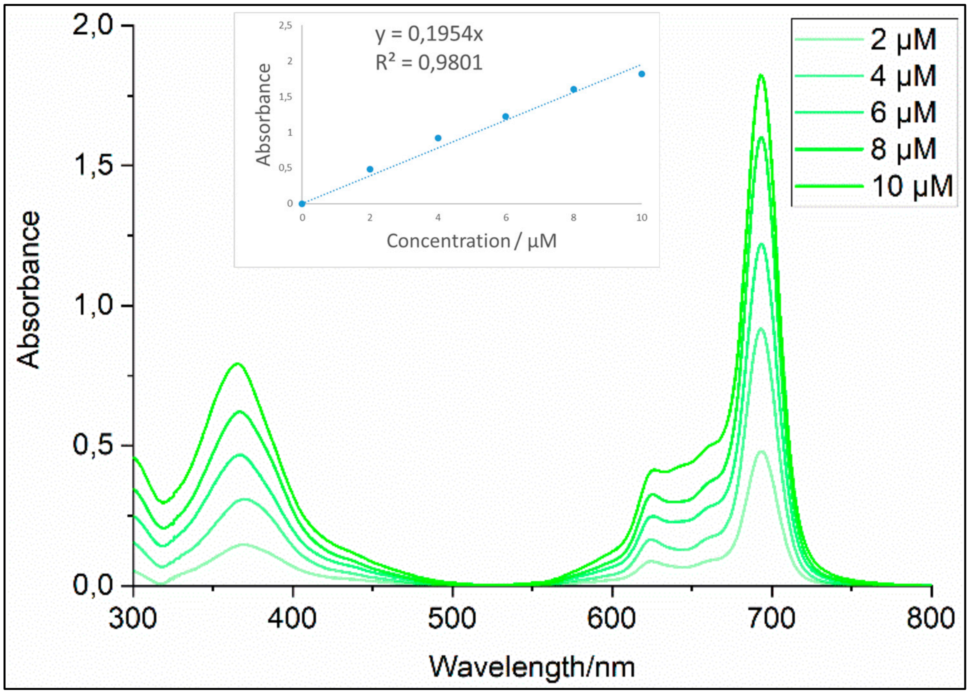

Figure S2. UV-vis spectrum of **$ZnPc(SPr)_8$** in DMF (2-10 µM).

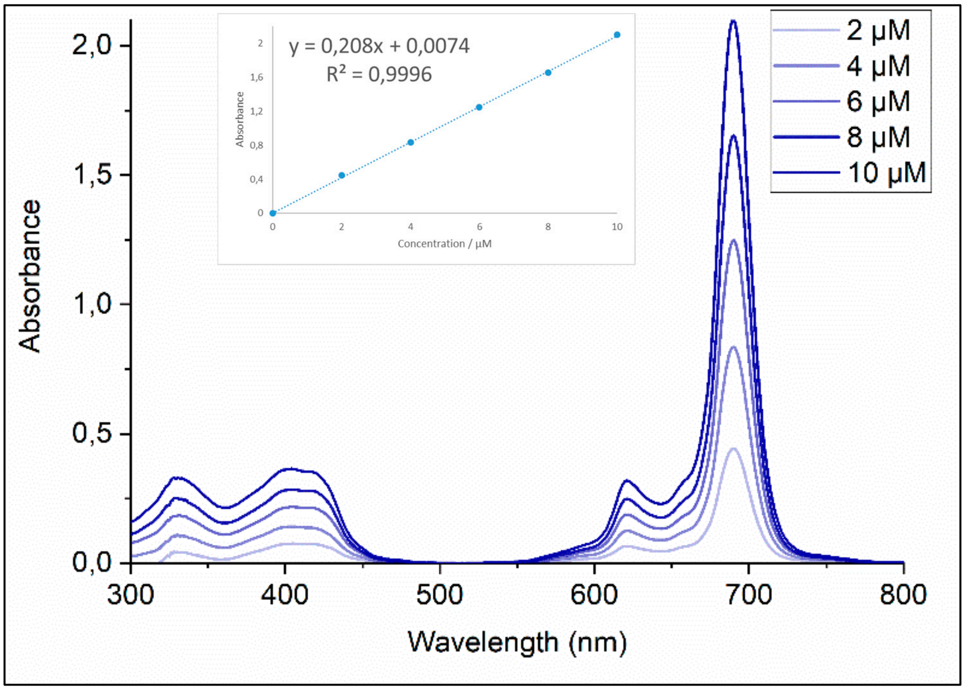

Figure S3. UV-vis spectrum of **ZnPc(SO$_2$Pr)$_8$** in DMF (2-10 μM).

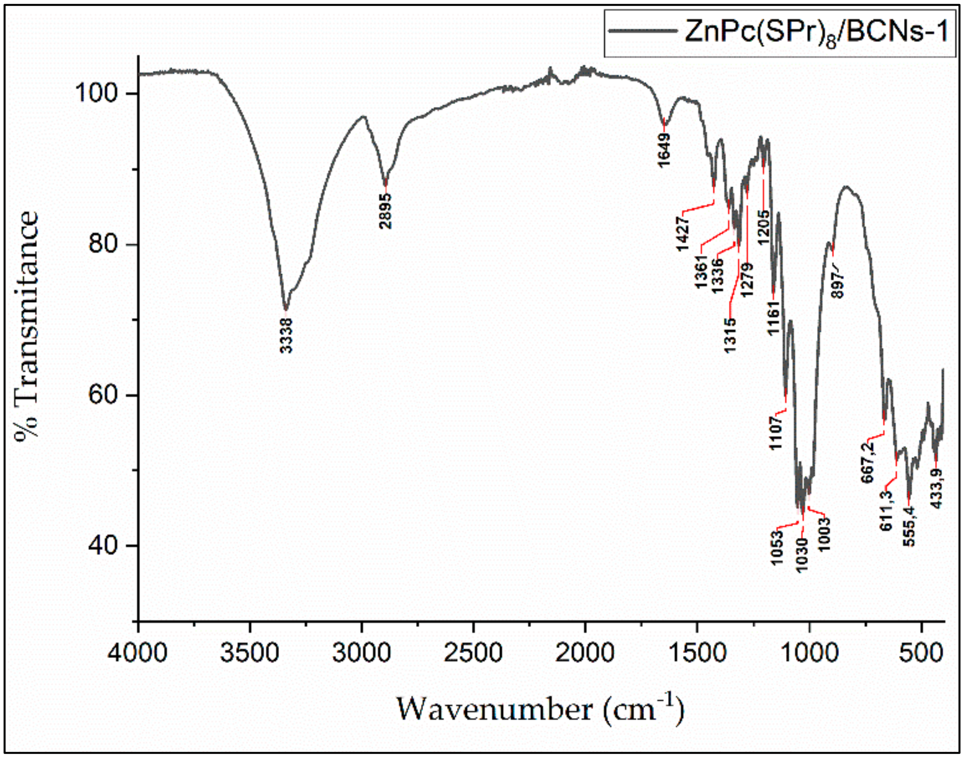

Figure S4. FT-IR spectrum of **ZnPc(SPr)$_8$/BCNs-1**

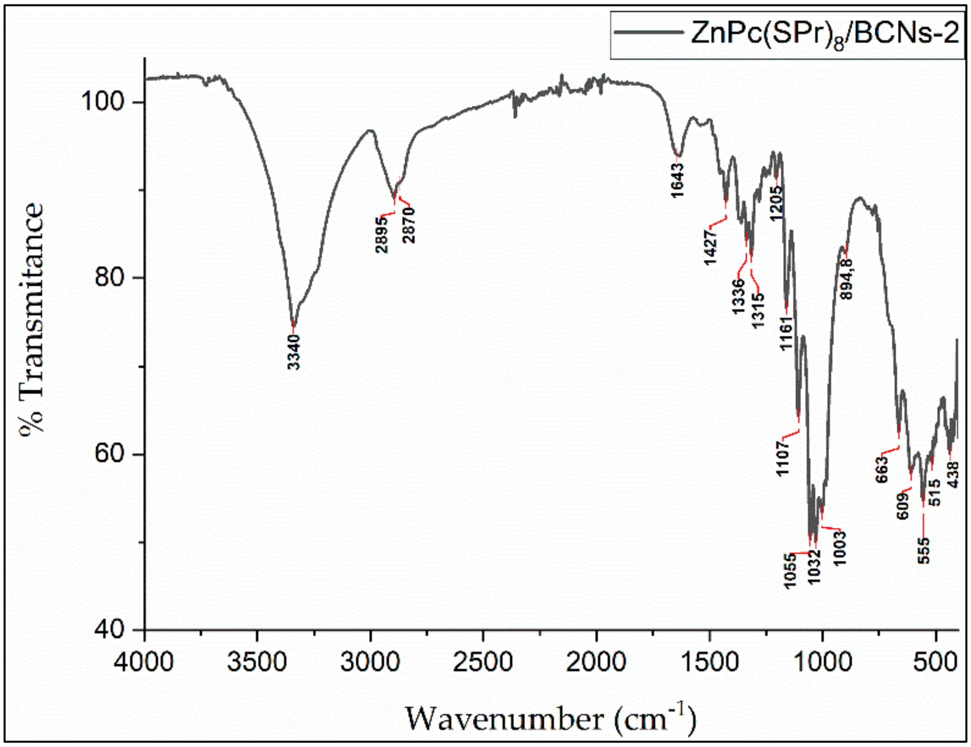

Figure S5. FT-IR spectrum of **ZnPc(SPr)$_8$/BCNs-2**

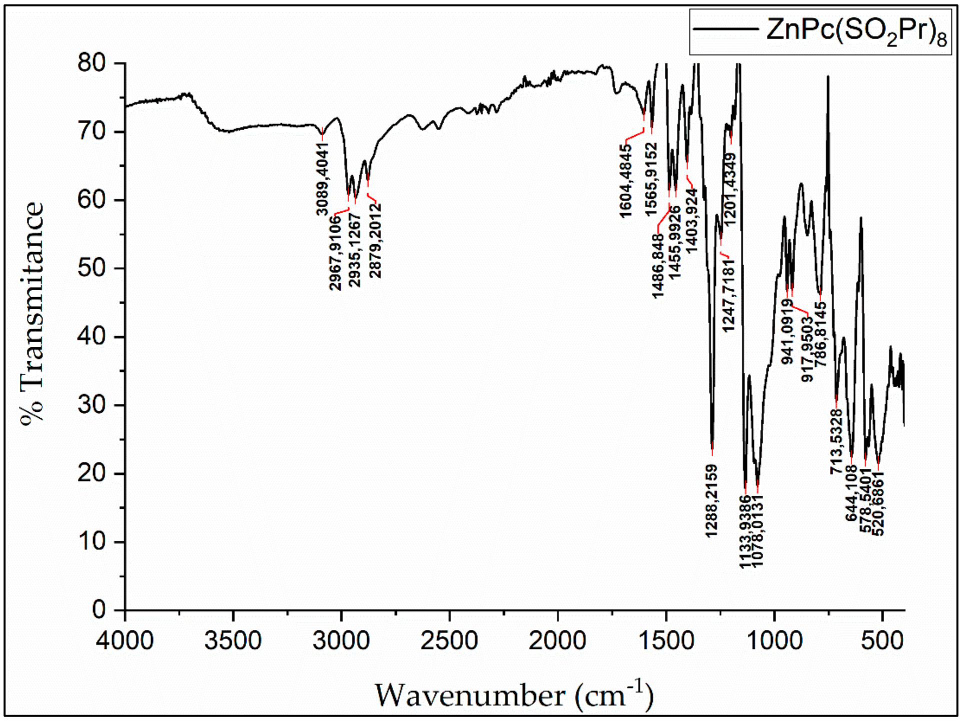

Figure S6. FT-IR spectrum of the **ZnPc(SO$_2$Pr)$_8$**.

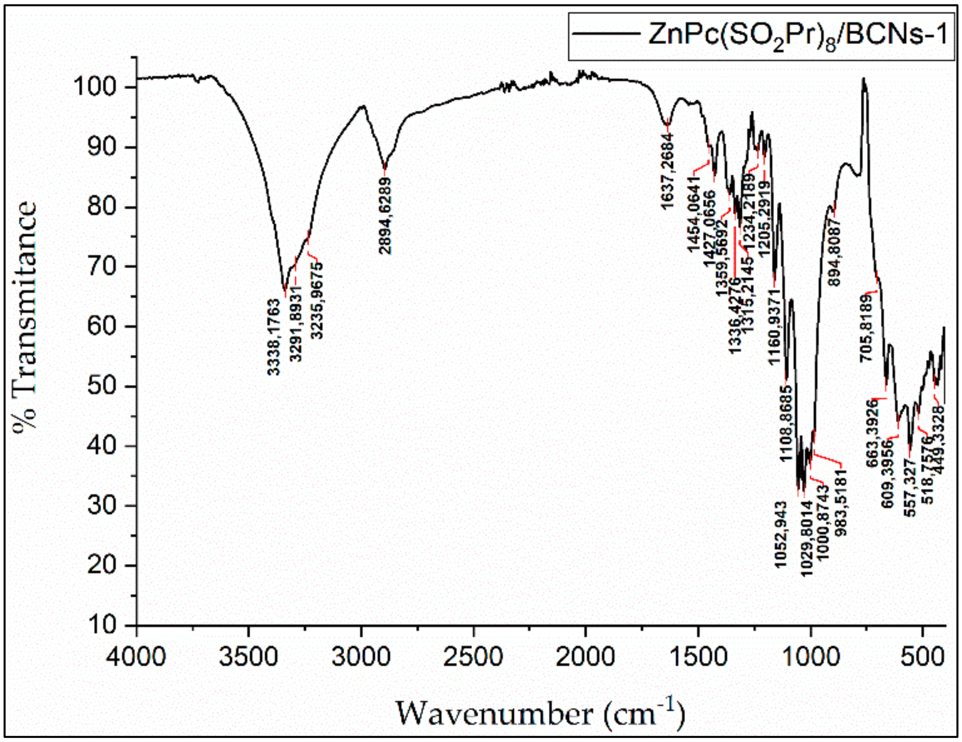

Figure S7. FT-IR spectrum of **ZnPc(SO$_2$Pr)$_8$/BCNs-1**

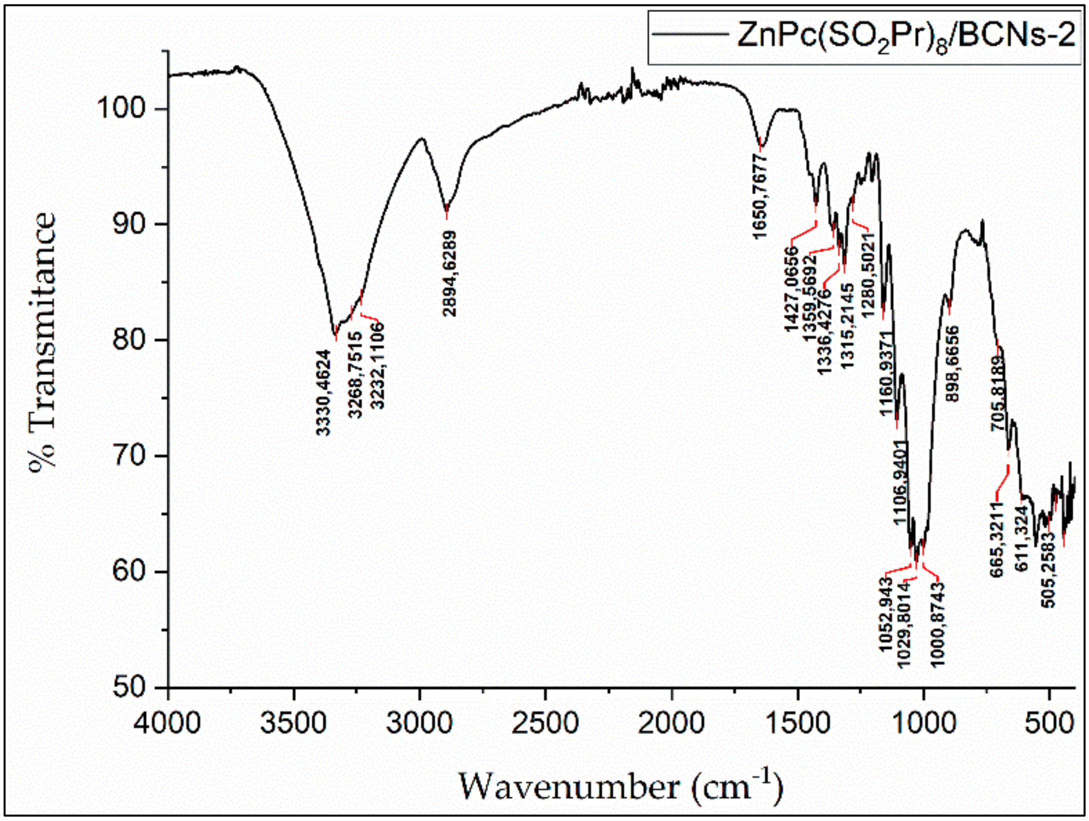

Figure S8. FT-IR spectrum of **ZnPc(SO$_2$Pr)$_8$/BCNs-2**.

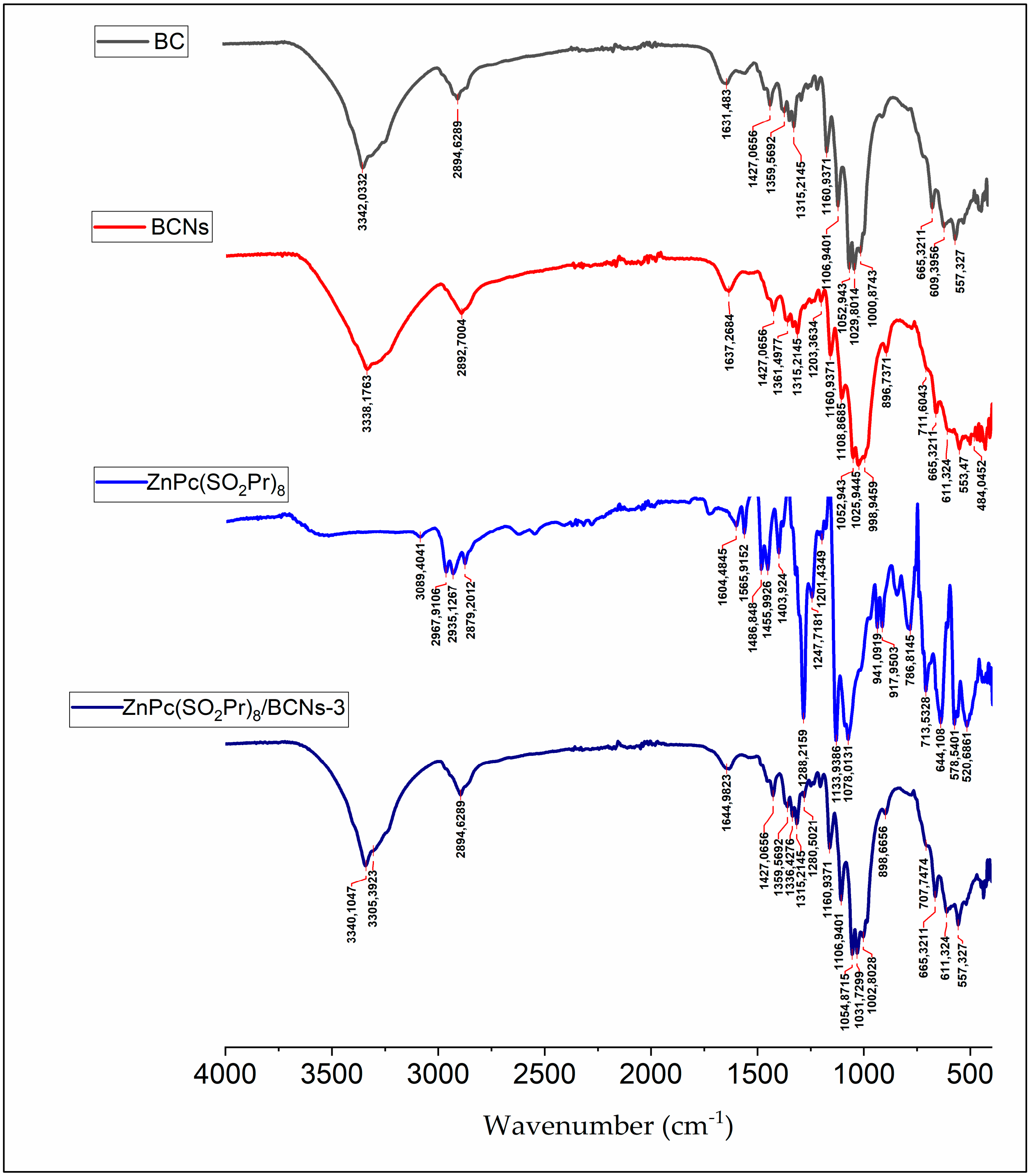

Figure S9. Superimposed FT-IR spectra of BC, BCNs, **ZnPc(SO_2Pr)$_8$** and **ZnPc(SO_2Pr)$_8$/BCNs-3**.

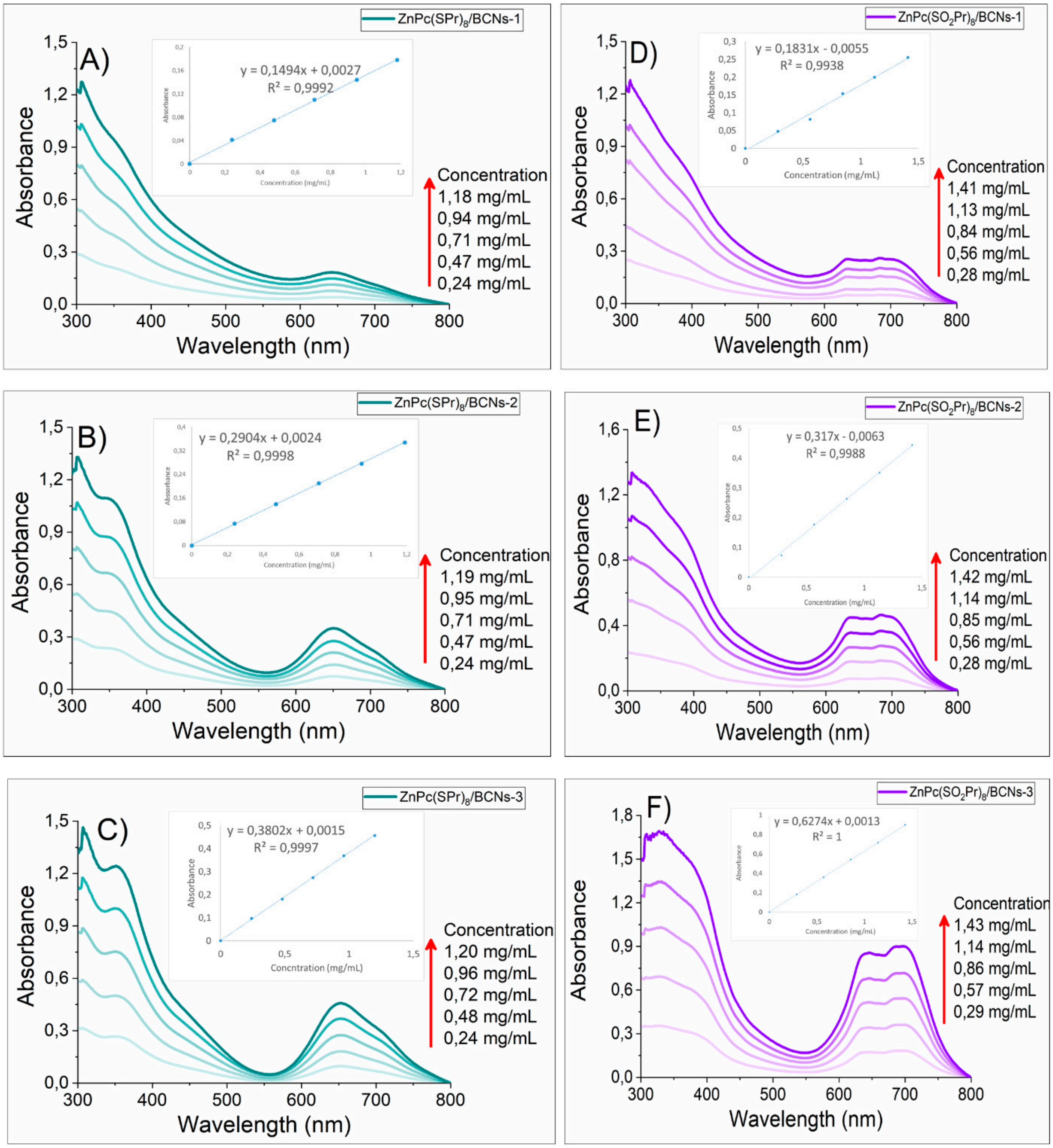

Figure S10. UV-vis spectra of the Pc/BCNs samples at different concentration (mg/mL) in water.
